# New blueberry and *mortiño* relatives (Ericaceae) from northwestern Colombia

**DOI:** 10.3897/phytokeys.49.8383

**Published:** 2015-04-22

**Authors:** Paola Pedraza-Peñalosa

**Affiliations:** 1 Institute of Systematic Botany, The New York Botanical Garden, 2900 Southern Blvd., Bronx, NY 10458

**Keywords:** *Psammisia*, *Satyria*, Vaccinieae, Chocó biogeographic region, Tropical Andes, Las Orquídeas National Park

## Abstract

The inventory of the vascular plants of one of the richest and least studied floras, the Andean and Chocó regions of northwestern Colombia, targets Las Orquídeas National Park. As a result of field trips to areas never before collected, several epiphytic and small terrestrial shrubs in the family Ericaceae have been discovered in the Park’s humid forests. Five new, morphologically remarkable species of Ericaceae (tribe Vaccinieae), are here described and illustrated. In a separate phylogenetic analysis, *Psammisia pinnata* Pedraza, *Psammisia pseudoverticillata* Pedraza, *Satyria orquidiensis* Pedraza, and *Satyria pterocalyx* Pedraza, were placed by molecular sequence data within clades of the non-monophyletic genera *Psammisia* and *Satyria*; phylogenetic evidence for the placement of *Psammisia sophiae* Pedraza is still lacking. Their affinities are here discussed, along with their preliminary conservation status.

## Abstract (es)

El inventario florístico de las plantas vasculares de una de las floras más ricas y menos estudiadas, los Andes y Chocó del noroccidente colombiano, se centra en el Parque Nacional Natural Las Orquídeas. Como resultado de trabajo de campo en áreas del parque nunca antes colectadas, se han descubierto varias especies epífitas y arbustivas de la familia Ericaceae, las cuales habitan los bosques húmedos de la reserva. Cinco nuevas especies de Ericaceae (tribu Vaccinieae), con peculiar morfología, son acá descritas e ilustradas. En un análisis filogenético separado y basado en datos moleculares, *Psammisia pinnata* Pedraza, *Psammisia pseudoverticillata* Pedraza, *Satyria orquidiensis* Pedraza, y *Satyria pterocalyx* Pedraza, fueron asignadas a clados de los géneros no monofiléticos *Psammisia* y *Satyria*; para la asignación de *Psammisia sophiae* Pedraza no hay evidencia filogenética. Tanto las afinidades, como el estado preliminar de conservación de las nuevas especies, son acá discutidos.

## Introduction

The Colombian Western Cordillera and adjacent Chocó region, which belong to the Tropical Andes and Chocó biodiversity hotspots (Mittermeier et al. 1998, Myers et al. 2000), respectively, have the highest angiosperm diversity in N South America (Morawetz and Raedig 2007). Despite their importance, entire lineages endemic to or particularly rich in western Colombia are missing in monographic and phylogenetic works, as collections from this region have not been readily accessible, especially outside of the country. This unfortunate situation has silently weakened the scope of botanical research, while also undermining strategic planning for conservation and development in the Tropical Andes and the Chocó biogeographic regions, the world’s first and fifth priority areas for conservation (Churchill et al. 1995, Mittermeier et al. 1998, Mast et al. 1999, Myers et al. 2000, Orme et al. 2005).

To bring attention back to one of the richest and least studied floras, an inventory of the vascular plants of Las Orquídeas National Park (LONP) was undertaken. LONP is strategically located in the confluence of the Tropical Andes and Chocó, in the Department of Antioquia (Colombia). Nested in the western slope of the Western Cordillera, LONP (29,118 ha; practically the same size of Grenada) boast an enormous altitudinal gradient (ca. 300–3,450 m) and consequent range of vegetation. In general, the forests below 2,000 m are considered to be part of the Chocó phytogeographic region (Rangel-Ch. et al. 2004) while the remainder is Andean.

The ongoing inventory of the vascular plants of LONP particularly targets remote areas that have never been botanically explored before. In addition to trees, collecting is also focused on non-tree plants, especially those that are epiphytic and which are usually omitted from rapid floristic and vegetation assessments in the tropics. This holistic collecting strategy has resulted in the discovery of several new non-tree plant species, including five members of the family Ericaceae, all placed in the berry producing tribe Vaccinieae. These five new species are all restricted to the Colombian Western Cordillera and Chocó biogeographic region, and three of them are endemic to LONP. The new species are here described, illustrated, and discussed. Their preliminary conservation status is also included, based on the author’s field experience and knowledge of Colombian herbarium collections worldwide.

## Vaccinieae diversity in Colombia

Although Vaccinieae is geographically widespread, the greatest species diversity lies in the mountains of Papua New Guinea and the Neotropics, most prominently in the N Andes. In the Neotropics, there are 46 putative genera and more than 800 species of Ericaceae and the great majority belong to the inferior-ovaried tribe Vaccinieae (Luteyn 2002); actually, 27 genera are native to the Neotropics and all are Vaccinieae. The members of Vaccinieae are better known by the edible and commercially important blueberry, from North America, and the *mortiño* [*Vaccinium floribundum* Kunth], from South America.

In the Neotropics, the most extensive radiation of Ericaceae took place in Colombia with 24 genera and 278 species documented, that is ca. 35 % of the neotropical Ericaceae. Notably, about 55 % of the Colombian species are endemic to the country (Pedraza-Peñalosa unpubl.). Within Colombia, the greatest diversity is found in Antioquia, where 18 genera and at least 116 species were recently documented (Pedraza 2011), before the discovery of the five new species from LONP here described. Although Colombia contains already described hyper-diversity, its Ericaceae flora still remains poorly known, with many suspected undescribed species from sea level to the páramo.

## Placement and phylogenetic affinities of new species

The taxonomic placement of new Vaccinieae is difficult due to the striking disparity between generic-level phylogenetic relationships and current classification systems (Kron et al. 2002, Powell and Kron 2003, Pedraza-Peñalosa 2009, 2010). Therefore, to determine the generic placement of the new species, four were included in a comprehensive phylogenetic analysis of neotropical Vaccinieae, based on chloroplast and nuclear sequence data of 91 species (Pedraza-Peñalosa et al. 2015). Unfortunately, amplifications of the fifth new species (*Psammisia sophiae* Pedraza), which bears the most complex corolla structure, were not successful.

The molecular dataset was particularly rich in species of *Satyria* Klotzsch and *Psammisia* Klotzsch, both non-monophyletic and broadly defined groups, to which the five new species are initially assigned based on their overall morphology (Pedraza-Penalosa et al. 2015). However, the new species possess unusual characters for the genera in which they are provisionally placed: *Psammisia sophiae* has a corolla morphology unique among neotropical Vaccinieae; *Psammisia pinnata* Pedraza is among the few *Psammisia* s.l. with very large and pinnate leaves; *Psammisia pseudoverticillata* Pedraza is the only species in the group with clustered leaves and one of the few with markedly angled calyces; *Satyria orquidiensis* Pedraza is the only species in the group with clustered leaves, while its calyx ribs and constrictions makes it one of two species of *Satyria* s.l. with ornamented calyces; and lastly, *Satyria pterocalyx* Pedraza is the only other species in the group with ornamented calyx and apparently the only one with a corolla throat both dramatically constricted and elongated into a tube.

Eighteen out ca. 24 currently recognized species of *Satyria* s.l. were analyzed. The resulting best ML tree unequivocally placed the two new species described here, *Satyria orquidiensis* and *Satyria pterocalyx*, within a group that comprises *Satyria* s.s. (Pedraza-Peñalosa et al. 2015). Despite the fact that *Satyria orquidiensis* and *Satyria pterocalyx* are endemic to the same region, have similar corolla colors, and are the only *Satyria* s.s. known to have an ornamented calyx (winged and/or lobed), they are not closely related to each other.

The non-monophyletic *Psammisia* was split in the molecular analysis (Pedraza-Peñalosa et al. 2015). The largest of the groupings, *Psammisia* I clade, was dominated by species from the northern Andes. *Psammisia pinnata* and *Psammisia pseudoverticillata* were both placed within one of its subclades, one mostly composed of Colombian species. All the species in this subclade share chartaceous to subcoriaceous leaves with pinnate venation (the majority of *Psammisia* have plinerved and coriaceous leaves) and short racemes with a rachis typically less than 1.6 cm long, which give inflorescences a fasciculated appearance, their corollas are medium size (8–22 mm long). However, the molecular sequence data show that the newly described *Psammisia* are not closely related.

Unfortunately, at this time it is not known which clade in the *Psammisia* complex will retain the generic name, as *Psammisia falcata* (Kunth) Klotzsch, the type species of the genus has not been sequenced yet. Thus, taxonomic and nomenclatural changes in *Psammisia* s.l. are anticipated. Moreover, because only 14 out of about 70 spp. of *Psammisia* species were analyzed, a broader sampling of the group and related genera is needed to better infer the relationships of the new species described here. Names are coined here in order that they may be used in future studies both of phylogeny and conservation of Colombian plants.

### New species from Las Orquídeas National Park (Colombia)

#### Psammisia pinnata

Taxon classificationPlantaeEricalesEricaceae

Pedraza sp. nov.

urn:lsid:ipni.org:names:77146695-1

Figures 1 , 2 , 3

##### Diagnosis.

*Psammisia pinnata* stands out among all other *Psammisia* s.l. because its distinctive large leaves (among the largest in the genus) that are subcoriaceous to chartaceous, elliptic to oblong, sometimes slightly asymmetrical, and which have pinnate venation; its petioles are pulvinate. Also distinctive is its ridged bark and conspicuous raceme rachises, and the relatively long flowers with staminal filaments fused at their very base.

##### Type.

COLOMBIA. Antioquia: Municipio Urrao. Corregimiento La Encarnación, Parque Nacional Natural Las Orquídeas, camino entre el páramo del Almorzadero y la cabaña de Calles, [6°31'N; 76°15'W. 1400 m], 31 Jul 2011 (fl, fr) *P. Pedraza-Peñalosa, J. Betancur, M. F. González, R. Arévalo, D. Sanín, A. Zuluaga, A. Duque & J. Serna 2491* (holotype: COL!; isotypes: HUA!, E!, MO!, NY! [NY02058401]).

##### Description.

Terrestrial or epiphyte *shrubs*, more or less erect or with arching branches, 1.5 m tall; stems brown-black, ridged or subterete, with soft and small grooves twisting near nodes, glabrous, pith drying dark purple, most terminal branches usually hollow, and, at least in one occasion, inhabited by ants; twigs with a few deep ridges running lengthwise and often twisting near nodes, glabrous. Axillary buds compressed; prophylls 2, inconspicuous, valvate, ovate, 1.5–2 mm long, margin ciliolate, the hairs unicellular and eglandular (all indumentum composed of this type of hairs except when indicated), apex acute, glabrous. *Leaves* alternate; petiole subterete, thick and basally pulvinate, 1–2.8 cm long, glabrous; lamina subcoriaceous to chartaceous, elliptic to oblong, sometimes slightly asymmetrical (more evident in large leaves), (19–)23–45 × (5–)8.7–22 cm, base cuneate to sometimes nearly truncate, margin entire and eciliate except for the very young leaves with a handful of caducous apical hairs, apex (long or short) acuminate, glabrate with caducous hairs on both sides, adaxial hairs inconspicuous (< 0.5 mm long), abaxial hairs often affixed atop of what seem to be minute laminar glands; laminar glands only evident abaxially, drying black, sparse, small, and round; venation pinnate, with up to 5 visible orders in dry specimens, 8–11 secondaries per side, these alternate (rarely subopposite), evenly dispersed along the lamina, ascending, brochidodromous, intersecondaries present toward midsection, midrib and secondaries adaxially impressed and abaxially raised, tertiaries well marked (in mature leaves), parallel among themselves and inserted at ca. 80–90° with respect to midrib. *Inflorescence* an axillary, solitary, 5–11-flowered raceme, often cauliflorous; inflorescence bracts caducous, chartaceous, ovate, 1.2–1.8 × 1.7–3 mm, margin entire and eciliate, apex obtuse, glabrous on both sides, venation obscure; rachis pink or magenta (fuchsia), 9–16 mm long, glabrous; floral bract 1, persistent, chartaceous, white, ovate, 1–3 × 1.5–2.5 mm, margin entire and eciliate, apex obtuse to acute, glabrous on both sides, venation obscure; pedicel pink or magenta (fuchsia), articulated with calyx, 9–23 mm long, glabrous; bracteoles 2, persistent, medially to distally inserted, opposite, chartaceous, white, ovate, 1.3–1.6 × 1–1.2 mm, margin entire and eciliate and with one or two pairs of stout masses of fimbria, apparently glandular in nature, easily breakable, apex acute, glabrous on both sides, venation obscure. *Flowers* 5-merous, actinomorphic, diplostemonous. Calyx pink or magenta, the lobes whitish with black marginal glands, cupuliform and sometimes slightly flaring out apically (urceolate *in vivo*), 6–8.5 mm long, glabrous; tube cupuliform, terete, ca. 3.5 mm long; limb spreading when dry (erect *in vivo*), 3–4 mm long; lobes ovate, 1–1.8 × 2–3.5 mm long, glandular margin on each side of the lobe (excluding the apex) sometimes breaking into stout segments of fusing glandular fimbria (sensu Luteyn 1983), margin eciliate, apex acute; sinuses obtuse (U-shaped); aestivation unknown. Corolla white (in bud basally pink and distally white), fleshy, not bistratose, conic, terete, 17–22 mm long, ca. 4.5 mm diam., ca. 2.5 mm wide at throat, glabrous within and without; lobes ovate, 1.2–1.3 × 1.4–1.5 mm, apex acute; aestivation valvate. *Stamens* 10, equal (though one cycle very slightly shorter than the other by < 0.5 mm), 7–9 mm long, included, not adherent to corolla; filaments connate in basal 0.3–1.5 mm, 3–4.7 mm long, glabrous, marginally glabrous or glabrate, the hairs inconspicuous, ca. 0.1 mm long; anthers 7–8 mm long, connective spurless; thecae 4.5–5 mm long, prognathous, without basal appendage, papillate; tubules 2, free, straight, basally similar in width to thecae, 2.2–3 mm long, smooth, dehiscing by introrse slits almost as long as the tubules, 2.2–2.7 mm long. *Ovary* 5-locular; nectary not pulvinate, top of ovary flat or concave; style 17–20 mm long, included; stigma punctiform. *Berry* ca. 12 mm diam., turning green with age, the lobes yellowish and converging.

**Figure 1.**
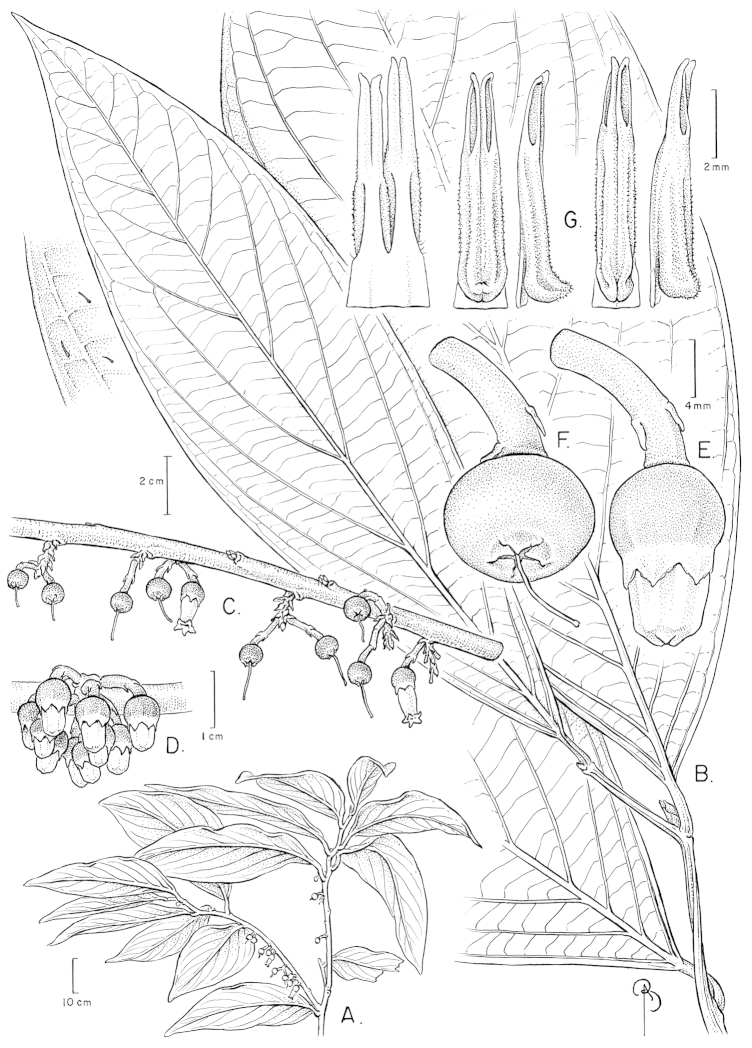
Illustration of *Psammisia pinnata*. **A** Branches with leaves and inflorescences; general aspect of the plant **B** Close-up of leaves with detail of hairs **C** Branch with inflorescences and flowers at and post anthesis **D** Immature inflorescence with floral buds **E** Immature flower with pedicel **F** Calyx post-anthesis **G** Stamens in lateral, abaxial, adaxial and views. [Drawn from the type and *P. Pedraza-Peñalosa et al. 2015*.]

**Figure 2.**
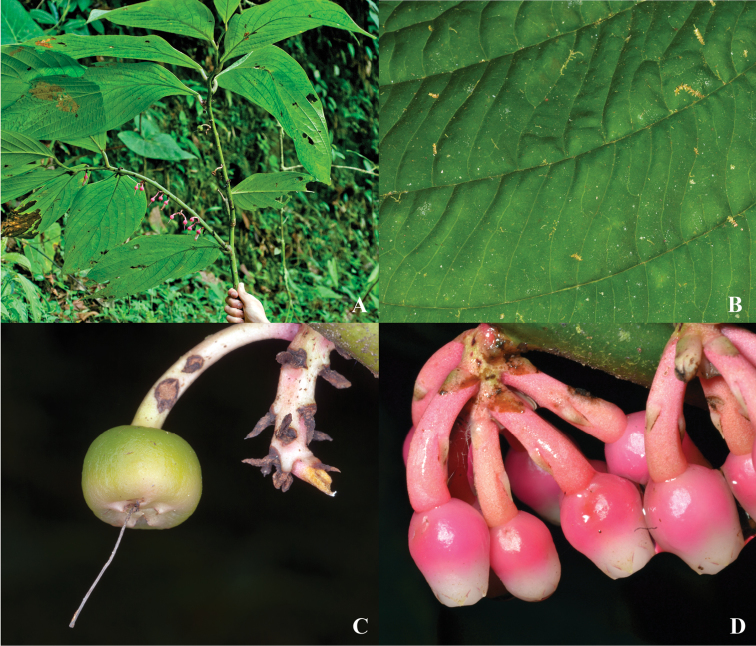
*Psammisia pinnata*. **A** Branches with leaves and inflorescences **B** Adaxial detail of leaf venation **C** Immature fruit attached to inflorescence axis **D** Floral buds, lateral view. [Photos by P. Pedraza-Peñalosa (**A–C**) and Nelson R. Salinas (**D**).]

**Figure 3.**
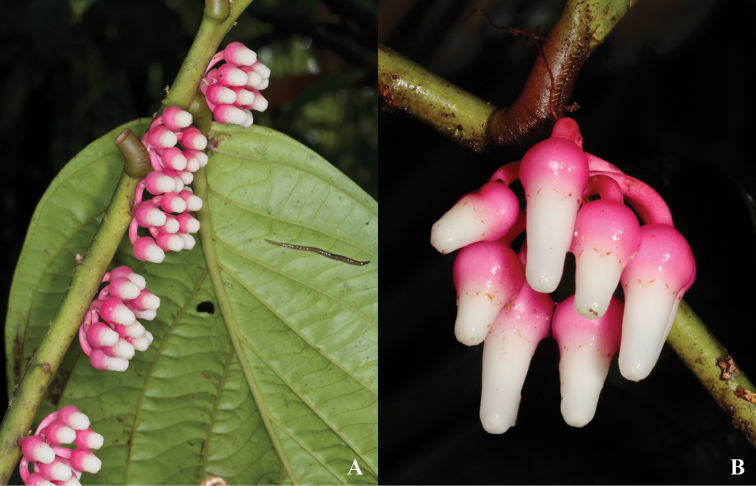
*Psammisia pinnata*. **A** Immature inflorescences and abaxial side of leaf **B** Immature flowers. [Photos by P. Pedraza-Peñalosa (**A**) and Nelson R. Salinas (**B**).]

##### Distribution and ecology.

*Psammisia pinnata* is restricted to the rich humid premontane and montane forests of the Colombian Western Cordillera (Antioquia, Risaralda, and Valle del Cauca) where it is known to flower and fruit in January, April, and July. It grows at 950–1900 m.

##### Etymology.

The species name indicates the characteristic pinnate leaf venation.

##### Preliminary conservation status.

*Psammisia pinnata* occupies a large geographic area and ample altitudinal range, therefore there is no concern for its conservation status. However, it must be noted that Colombian Andes have alarming rates of deforestation and transformation, putting its natural vegetation under constant threat.

##### Discussion.

*Psammisia pinnata* is perhaps morphologically close to *Psammisia multijuga* Sleumer although it is clearly differentiable because of the soft bark grooves that twist near the nodes (vs. bark smooth in *Psammisia multijuga*); dark purple branch pith (vs. white pith); elliptic to oblong (vs. ovate), apically acuminate (vs. abruptly acuminate [subcuspidato-acuminata]), and glabrous (vs. with inconspicuous hairs on both sides) leaves; black laminar glands evident (but small) abaxially (vs. few and inconspicuous); well-marked (abaxially) parallel tertiary venation, distinctively inserted at ca. 80–90° with respect to midrib (vs. not well-marked and reticulated); larger and fuller racemes with 5–11 flowers and rachises 9–16 mm long (vs. 2-flowered racemes and 2–4 mm long rachises [4–5-flowered fascicle in the protologue of *Psammisia multijuga*]); glabrous calyces (vs. inconspicuously puberulous, the hairs eglandular and <0.5 mm long [glabrous in the protologue of *Psammisia multijuga*]), with lobes with glandular margins (vs. eglandular); longer corollas (17–22 vs. 15 mm); basally connate staminal filaments, 3–4.7 mm long (vs. free, 2–2.5 mm long); tubules 2.2–3 mm long, dehiscing by slits almost as long as the tubules (vs. tubules 4.3–4.6 mm long, dehiscing by short slits 1.5 mm long).

##### Specimens examined.

**COLOMBIA. Antioquia:** Municipio Urrao, Corregimiento La Encarnación, Vereda Calles, Parque Nacional Natural Las Orquídeas, finca de Alfonso Pino, entre la divisoria de las quebradas La Virola y El Bosque, al NW de la cabaña Calles, 6°31'35"N; 76°15'50"W, 1450–1470 m, 27 Jan 2011 (fl, fr), *P. Pedraza-Peñalosa et al. 2015* [COL! (2 sheets), HUA, MO, NY! (2 sheets)]; Parque Nacional Natural Las Orquídeas, sector Calles arriba, sitio El Macho, 6°32'22"N; 76°14'05"W, 1700–1750 m, 9 Dec 2013 (fl), *N. R. Salinas et al. 865* [COL, E!, HUA, LPB!, MO!, NY! (2 sheets)]; Near top of Cordillera Occidental on trail from Encarnación to Parque Nacional Natural Las Orquídeas, 1900–2100 m, 27 Jan 1979 (fl), *A. Gentry & A. Renteria 24641* (COL, MO!, NY!); Corregimiento Nutibara, region of Murrí, Nutibara-La Blanquita road, 1700–1800 m, 19 Apr 1988 (fl. buds), *J. L. Luteyn et al. 12002* (AAU, COL, HUA, MO, NY!), 950–1380 m, 20 Apr 1988 (fl. buds), *J. L. Luteyn et al. 12110* (COL, NY!). **Risaralda:** Municipio Mistrató, Inspección de Policia de Jeguadas, camino entre Jeguadas y Puerto de Oro, entre los sitios Curramaí y Pisones, 1200–1500 m, 3 Abr 1992 (fr), *J. Betancur et al. 3312* (COL, NY!). **Valle del Cauca:** Municipio Cali, Finca Zingara, km 18 de la carretera Cali-Buenaventura, km. 4 vía Dapa, Corregimiento La Elvira, 1900 m, 2 Abr 2000 (fl), *J. Giraldo-Gensini 903* (NY!).

#### Psammisia pseudoverticillata

Taxon classificationPlantaeEricalesEricaceae

Pedraza sp. nov.

urn:lsid:ipni.org:names:77146696-1

Figures 4 , 5 , 6

##### Diagnosis.

*Psammisia pseudoverticillata* can be easily differentiated from all other *Psammisia* s.l. by its leaves, which are clustered and seemingly verticillate, chartaceous, large, obovate, bullate *in vivo*, pinnate, decurrent at the base, and subtended by a basally pulvinated petiole. Its congested racemes bear flowers that are distinctive because of their large size, thickness and fleshiness; however, it must be noted that the flowers of this new species shrink significantly upon drying. The flowers of *Psammisia pseudoverticillata* are also characterized by its color combination and angled calyces and corollas (calyces markedly angled).

##### Type.

COLOMBIA. Antioquia: Municipio Urrao. Corregimiento La Encarnación, Vereda Calles, Parque Nacional Natural Las Orquídeas, camino Calles-La Encarnación, después de la confluencia del Río Polo y el Río Calles, antes del Río San Pedro, sitio La Quiebra, 6°30'31"N; 76°14'W, 1600–1850 m, 31 Jan–2 Feb 2011 (fl), *P. Pedraza-Peñalosa, J. Betancur, M. F. González, G. Giraldo, F. Gómez, A. Duque & J. Serna 2134* (holotype: COL!; isotypes: HUA!, NY! [NY02058402]).

##### Description.

Terrestrial *shrubs* with arching branches, < 1 m tall; stems and twigs somehow flatten, caniculated lengthwise, apparently glabrous, the hairs inconspicuous (< 0.1 mm long), unicellular and eglandular (= minute hair type), bark brown and smooth. Axillary buds not observed. *Leaves* alternate, originated very close together, pseudoverticillate with clusters of 3–5 leaves separated by leafless sections several centimeters long; petiole caniculate, basally pulvinate, 4–12 mm long, glabrate, minute type of hair; lamina chartaceous, bullate *in vivo*, obovate, (17.5–)20.5–24 × (6.5–)12–14.2 cm (at least 24 cm long, apices incomplete), base attenuate and decurrent, margin entire and eciliate, apex missing in herbarium specimens but probably acuminate, glabrate on both sides, adaxially with caducous minute hairs, abaxially the hairs of arachnoid type, ca. 1 mm long, very thin, multicellular and eglandular; laminar glands absent; venation pinnate, with up to 4 orders visible adaxially in dry specimens, midrib adaxially impressed and abaxially raised, at least 9–11 secondaries per side, these alternate or subopposite, evenly dispersed along the lamina, ascending, adaxially flat and abaxially raised, brochidodromous, intersecondaries frequent. *Inflorescence* an axillary, solitary, 6–8-flowered raceme; inflorescence bracts, floral bract, and bracteoles alike, persistent, chartaceous, cream-reddish-colored, ovate, 1.6–1.8 × 1.5–2 mm, margin entire and inconspicuously ciliolate, the hairs of the minute type but a few are multicellular, apex obtuse or acute, glabrous on both sides, venation obscure, the bracteoles are different in having apex acute and more abundant marginal multicellular hairs that are fused in masses of fimbria toward the base; peduncle 4 mm long, rachis 5–10 mm long, both red-brown, glabrate, the hairs of the arachnoid type, also covered with minute, whitish warts *in vivo* (inconspicuous when dried, though imparting rough look); pedicel red-brown, articulate with calyx, 12–14 mm long (17 mm *in vivo*), with the same indumentum and warts of the rachis; bracteoles 2, basal, supopposite to alternate. *Flowers* 5-merous, actinomorphic, diplostemonous. Calyx red-brown, ellipsoid, 5-angled, the angles sharp and opposite to the sinuses, 8–9.2 mm long (10.5–11 mm *in vivo*), sparsely tomentulose with a combination of arachnoid and minute hair types, specially warty distally; tube ellipsoid, 6–7.4 mm long (8.5–9 mm *in vivo*); limb erect, 1.8–2 mm long; lobes deltate, 1 × 3.2 mm long, (1.5–1.7 × 4 mm *in vivo*), margin scariose (except at apex) and sometimes broken up in segments or with a few multicellular and eglandular hairs, apex acute; sinuses obtuse (U-shaped); aestivation unknown. Corolla dark pink with white throat and lobes, very fleshy, not bistratose, urceolate, 5-angled, 12–14 mm long (19 mm *in vivo*), 4–5.2 mm diam. (ca. 10 mm *in vivo*), 2.4–3 mm wide at throat (ca. 5 mm *in vivo*), puberulous without with a combination of arachnoid and minute hairs, the indumentum more abundant distally, glabrous within; lobes ovate, ca. 1.1 × 1.1 mm (ca. 2 × 2 mm *in vivo*), apex acute, reflexed at maturity; aestivation valvate. *Stamens* 10, equal, 8.7–9 mm long, included, not adherent to corolla; filaments free, long-triangular, 2.3–2.5 mm long, marginally glabrate, with minute hairs; anthers 8.3–8.5 mm long, connective spurless; thecae 4.1–4.5 mm long, slightly prognathous, without basal appendage, papillate; tubules 2, free, straight, 4–4.2 mm long, smooth, dehiscing by introrse slits, 2.7–3.5 mm long. Nectary not pulvinate, slightly concave *in vivo*, glabrous; style 15–17 mm long, included; stigma punctiform. *Berry* unknown.

**Figure 4.**
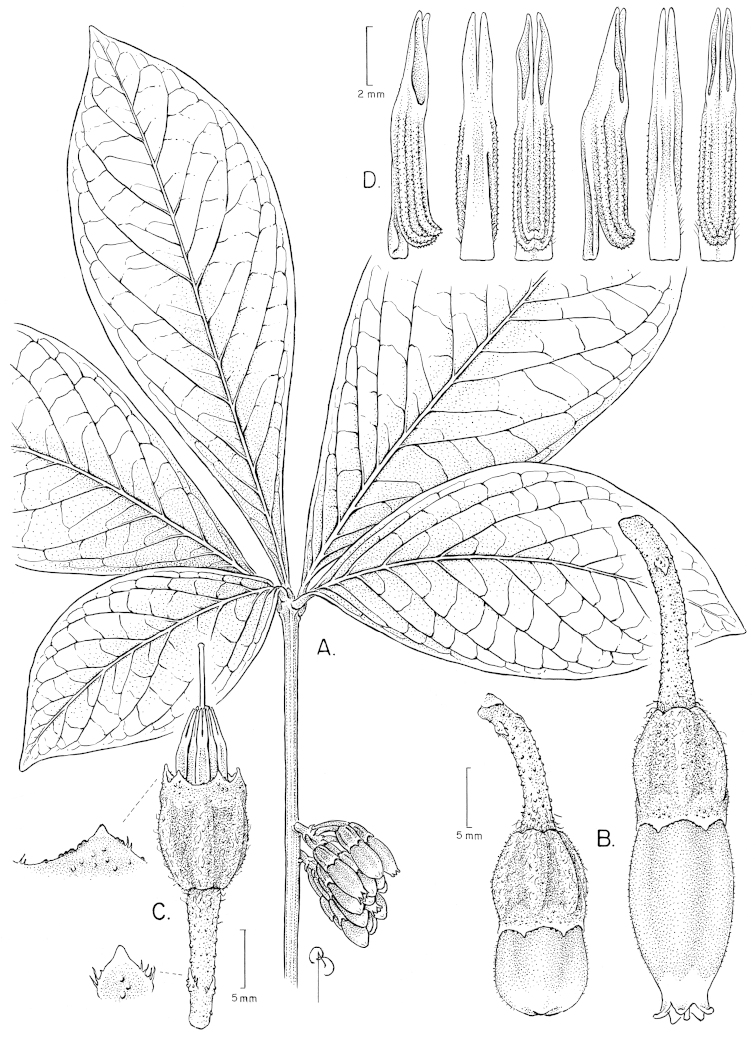
Illustration of *Psammisia pseudoverticillata*. **A** Branch with clustered leaves and inflorescence **B** Floral bud and mature flower **C** Flower with the corolla removed to show the stamens arranged around the style; details of the calyx lobes (above) and bracteoles (below) **D** Stamens in lateral, abaxial, adaxial and views. [Drawn from the holotype.]

**Figure 5.**
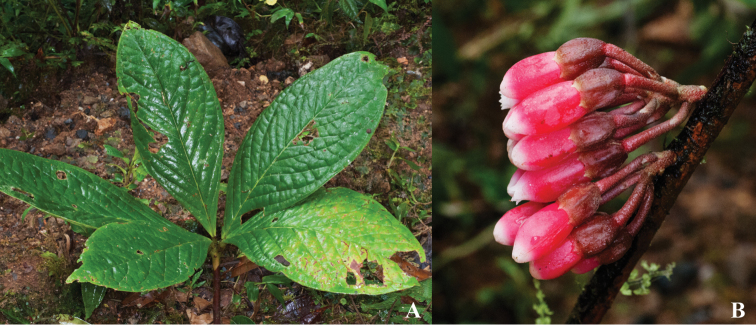
*Psammisia pseudoverticillata*. **A** Clustered leaves **B** Inflorescences, side view. [Photos by P. Pedraza-Peñalosa.]

**Figure 6.**
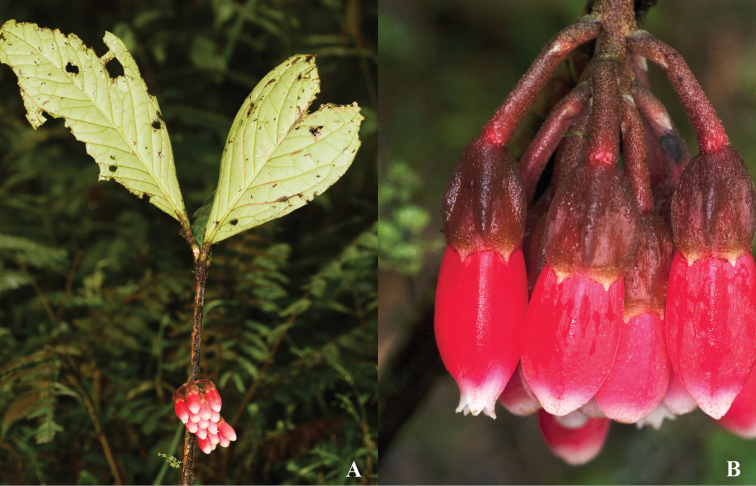
*Psammisia pseudoverticillata*. **A** Branch with leaves and inflorescences **B** Close up of flowers showing the angled calyces. [Photos by P. Pedraza-Peñalosa.]

##### Distribution and ecology.

*Psammisia pseudoverticillata* is endemic to Antioquia (Colombia) and it is only known by the type specimen collected in Las Orquídeas National Park. It is known to flower in January and February. It grows in humid montane forests at 1600–1850 m.

##### Etymology.

The species epithet refers to the clustered tendency of the leaves.

##### Preliminary conservation status.

*Psammisia pseudoverticillata* it is only known by the type specimen collected in montane forests of Las Orquídeas National Park. Only one individual has been observed throughout several years of fieldwork. Currently, montane forest, and the park in general, suffer from degradation product of human activities (agriculture, selective logging, livestock), therefore I consider this species vulnerable due to the small area it occupies, its perceived scarcity, and current habitat threats.

##### Discussion.

Apparently, *Psammisia pseudoverticillata* is the only in the genus with seemingly verticillate leaves; only *Psammisia oppositiflora* Luteyn has opposite or subopposite leaves. *Psammisia pseudoverticillata* is morphologically closer to *Psammisia orthoneura* but the latter is differentiated because of its evenly distributed glabrous leaves (vs. leaves clustered, glabrate with minute hairs in *Psammisia pseudoverticillata*); obscurely articulate pedicels or apparently continuous with the calyx (vs. articulate); shorter (7–8 mm long), cupuliform, terete calyces (vs. 8–11 mm long, ellipsoid, with 5 well-defined angles); terete and shorter corollas (12–13 mm long vs. 5-angled, 12–19 mm long); and its staminal connectives with obsolete spurs (according to protologue) (vs. without spurs). A plant collected in La Serranía de los Paraguas (Valle del Cauca, Municipio El Cairo), where several Ericaceae novelties have recently been found (Pedraza-Peñalosa 2008), was identified by James L. Luteyn as Psammisia aff. orthoneura. This specimen (*Luteyn 12330*) may belong to *Psammisia pseudoverticillata*. However, the flowers are too immature to make an accurate identification given slight differences in leaf shape.

#### Psammisia sophiae

Taxon classificationPlantaeEricalesEricaceae

Pedraza sp. nov.

urn:lsid:ipni.org:names:77146697-1

Figures 7 , 8

##### Diagnosis.

*Psammisia sophiae* differs from other *Psammisia* s.l. because of its extraordinary flowers of complex morphology and unusual color combination. The distal half of the globose corolla has five pronounced projections or ribs, which in combination with the Christmas candy cane color pattern impart the species its distinctive and unique look.

##### Type.

COLOMBIA. Antioquia: Municipio Urrao, Corregimiento La Encarnación, Parque Nacional Natural Las Orquídeas, camino entre el páramo del Almorzadero y la cabaña Calles, [6°31'N; 76°15'W, 1400 m alt], 31 Jul 2011 (fl), *P. Pedraza-Peñalosa, J. Betancur, M. F. González, R. Arévalo, D. Sanín, A. Zuluaga, A. Duque & J. Serna 2490* (holotype: COL!; isotype: NY! [NY02058403]).

##### Description.

Terrestrial or epiphytic *shrubs*, more or less erect, 0.5–0.6 m tall; stems vinaceous and shiny, terete to subterete, glabrous, bark smooth; twigs subterete, glabrous. Axillary buds compressed; prophylls 2, inconspicuous, valvate, ovate, 1–1.2 mm long, margin eciliate, apex acute, glabrous. *Leaves* alternate; petiole caniculate (more so distally), pulvinate almost along entire length, 1.4–2.2 cm long, glabrous; lamina subcoriaceous, very smooth and flexible, elliptic, 19–34.5 × 4.5–12.5 cm, base attenuate and decurrent, margin entire and eciliate, apex acuminate, discolor *in vivo* with the abaxial side contrastingly light green, when dry the abaxial side is still lighter but with a dark marginal band, adaxially apparently glabrous, the hairs inconspicuous (< 0.5 mm long), unicellular and eglandular (all indumentum composed of this type of hair except when indicated), abaxially puberulous, the hairs with swollen bases that give them the aspect of minute punctuations; laminar glands absent; venation pinnate, with up to 3 visible orders in dry specimens, midrib adaxially raised and abaxially flat, 9–13 secondaries per side, these alternate or subopposite, evenly dispersed along the lamina, ascending, adaxially and abaxially slightly raised, brochidodromous. *Inflorescence* an axillary, solitary, 3–5-flowered raceme; inflorescence bracts, floral bract, and bracteoles alike, persistent, chartaceous, ovate, 1.2–1.4 × 1–1.5 mm, margin entire and eciliate, apex acute, glabrous on both sides, venation obscure; rachis magenta (fuchsia), 1.8–3 mm long, glabrous, a few inconspicuous warts at base; pedicel magenta (fuchsia), articulate with calyx, 7.5–13 mm long, glabrous although a few, small, glandular hairs at articulation; bracteoles 2, basal, opposite, only differing from other bracts in having an inconspicuous glandular margin. *Flowers* 5-merous, actinomorphic, diplostemonous. Calyx magenta (fuchsia) with the lobes whitish (more so in bud), cupuliform, 7.5–8 mm long (10.5–11 mm *in vivo*), glabrous; tube cupuliform, terete, 5.5–5.8 mm long (7.8–8.5 mm *in vivo*); limb erect, 2–2.2 mm long (2–3.2 mm *in vivo*); lobes deltate, 1.1 × 2.5 mm long (1.2–1.5 × 3.5–4 mm *in vivo*), with a thin glandular margin (excluding apex and sinuses) that is inconspicuous in dry specimens, margin eciliate, apex blunt acute; sinuses obtuse (U-shaped); aestivation unknown. Corolla fleshy, not bistratose, globose (but wider in the apical half), basal half terete, apical half with 5 deep, wide and blunt ribs that surpass the corolla lobes by 1.5–2 mm and which slightly connivent distally, the ribs opposite to corolla lobes, 4 mm long, 2 mm wide; basic corolla color dull white but magenta between the ribs, from their bases up to the lobes sinuses, and continuing along the very margin of the lobes, the color pattern is such that when observed from the top, the corolla seem to have stripes with a color combination that is reminiscent of a Christmas candy cane, total corolla length 10 mm (7.5 mm long in immature flowers), ca. 8 mm diam. (ca. 11 mm *in vivo*), ca. 2 mm wide at throat, glabrous within and without; lobes deltate, 1–1.5 × 1.5–1.6 mm, apex acute, slightly reflexed; aestivation valvate. *Stamens* 10, equal, ca. 5.5 mm long, included, not adherent to corolla; filaments free, triangular, 2–2.2 mm long, adaxially inconspicuously glabrate, abaxially glabrous; adjacent anthers differing moderately in width, ca. 5.2 mm long, the innermost with an incipient bump on each side of the connective; thecae 3.2–3.7 mm long, prognathous, without basal appendage, papillate; tubules 2, free, straight, 1.5–2 mm long, smooth, dehiscing by introrse slits almost as long as the tubules, 1.2–2 mm long. *Ovary* 5-locular; nectary not pulvinate or evident, top of ovary completely flat; style ca. 7 mm long, included; stigma punctiform. Immature *berry* green, 8 mm diam.; seeds numerous, isodiametric, black when dry, with mucilaginous coat; embryo apparently white.

**Figure 7.**
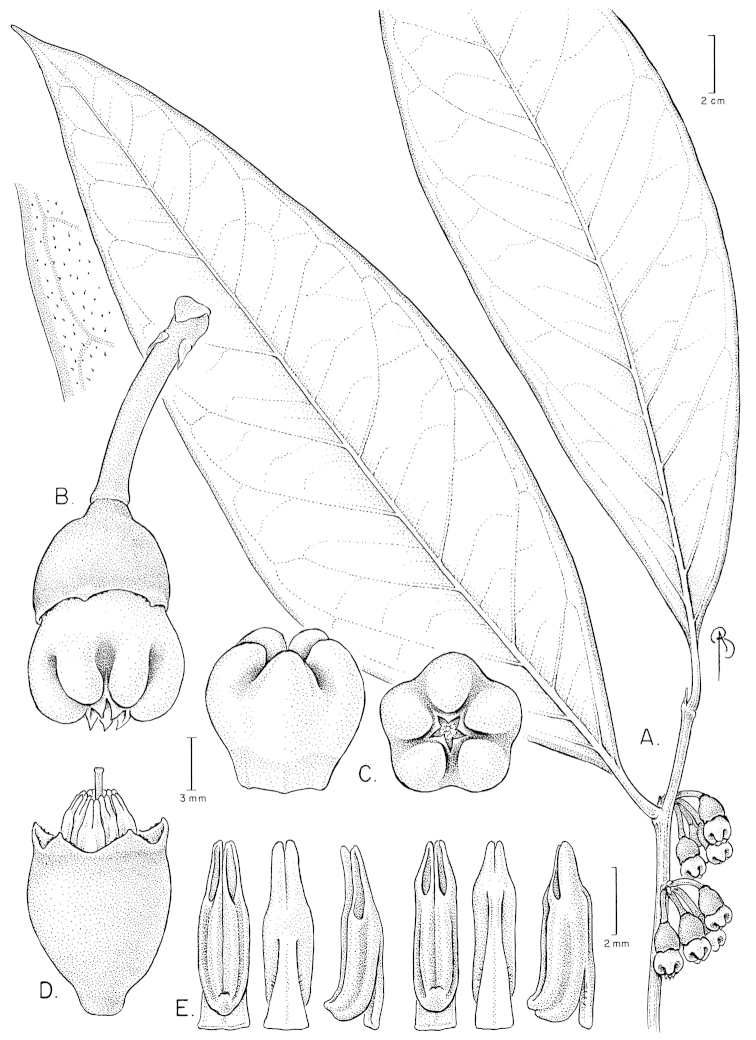
Illustration of *Psammisia sophiae*. **A** Branch with leaves, inflorescences and details of the darker colored leaf margin abaxially **B** Complete flower with pedicel and bracteoles **C** Lateral and top views of the corolla **D** Flower with the corolla removed to show the stamens arranged around the style **E** Stamens in abaxial, adaxial, and lateral views. [Drawn from the type.]

**Figure 8.**
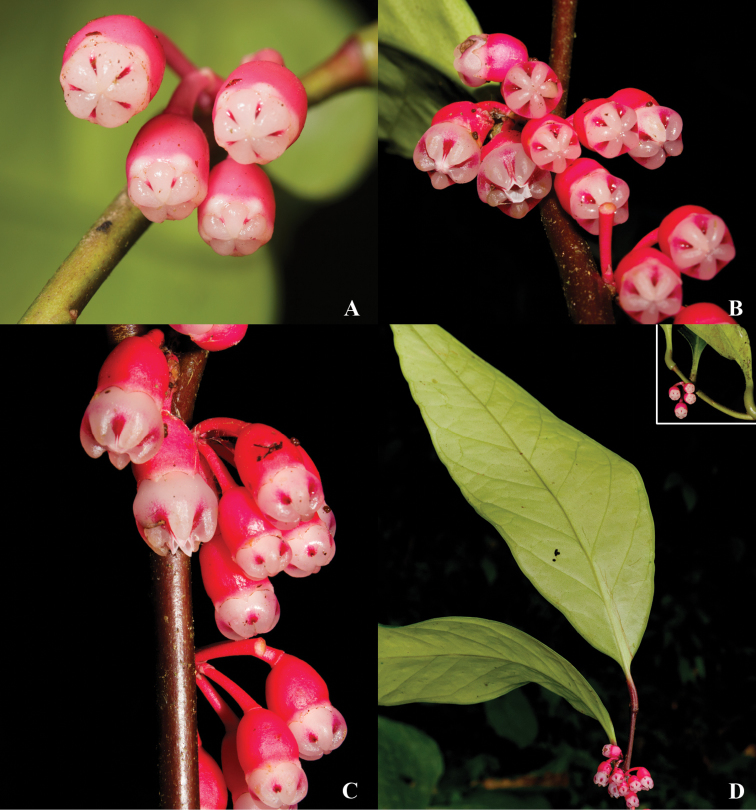
*Psammisia sophiae*. **A** Floral buds (top view) showing the distal ribs of the white corollas, which are magenta between the ribs **B** Top view of the inflorescence showing a flower at anthesis **C** Side view of the inflorescence showing a flower at anthesis **D** Branch with leaves, inflorescences and a detail of the pulvinate petioles. [Photos by P. Pedraza-Peñalosa.]

##### Distribution and ecology.

*Psammisia sophiae* is endemic to Antioquia (Colombia) and it has only been collected in Las Orquídeas National Park. It is known to flower in December and July and fruit in January. It grows in conserved humid premontane forests at 1160–1400 m.

##### Etymology.

Named after my daughter, Sofia Varón, an equally beautiful bloom.

##### Preliminary conservation status.

*Psammisia sophiae* is only known from collections made in Las Orquídeas National Park. Despite collecting in that protected reserve for many years only a few specimens have been procured. This species seems to prefer conserved premontane forests. Currently, these forest, and the park in general, specially toward lower elevations, suffer from degradation product of human activities (agriculture, selective logging, livestock). I consider this species vulnerable due to the small area it occupies, its perceived scarcity, and current habitat threats.

##### Discussion.

Although vegetatively similar, *Psammisia sophiae* can be told apart from *Psammisia panamensis*, not only because of their strikingly dissimilar flowers (obconic, terete, and with transversal bands of red, black and white in the latter), but also because the leaves of *Psammisia panamensis* are glabrous and when dried, the lamina has a black-bluish tint on both sides (vs. hairs present, lamina drying brownish with a distinctive dark marginal band abaxially in *Psammisia sophiae*), its petioles are not caniculate (vs. caniculate), and its rachis, bracts, bracteoles, and pedicel are longer.

##### Specimens examined.

**COLOMBIA. Antioquia:** Municipio Frontino, Parque Nacional Natural Las Orquídeas, Finca La Guadalupa, Quebrada Horacio, afluente del Río Venados, 1160 m, 1 Dec 1986 (fl), *R. Callejas et al. 2937* (NY!). Municipio Urrao, Corregimiento La Encarnación, Vereda Calles, Parque Nacional Natural Las Orquídeas, cabaña Calles, 6°31'09.1"N; 76°15'08.4"W, 1357 m, 25 Jan 2011 (fr), *P. Pedraza-Peñalosa et al. 1951* (COL!, MO!, NY!); Corregimiento La Encarnación, Vereda Calles, Parque Nacional Natural Las Orquídeas, finca de Alfonso Pino, entre la divisoria de las quebradas La Virola y El Bosque, al NW de la cabaña Calles, 6°31'35"N; 76°15'50"W, 1450–1470 m, 27 Jan 2011 (fl, fr), *P. Pedraza-Peñalosa et al. 2014* (COL!).

#### Satyria orquidiensis

Taxon classificationPlantaeEricalesEricaceae

Pedraza sp. nov.

urn:lsid:ipni.org:names:77146698-1

Figures 9 , 10 , 11

##### Diagnosis.

*Satyria orquidiensis* has many distinctive characters and it differs from other species in the genus because its leaves, which are clustered (up to 8 leaves) and seemingly verticillate (the only one in the genus), oblanceolate, large, and basally subcordate. The abundant and large flowers of this new species also stand out because of the big ribs on the calyx tube and the sharp transversal constriction between the limb and tube, which gives the entire calyx a broad campanulate shape. The long obconic corolla (up to 4.4 cm long) is orange and green at the tip. Both calyx and corolla are covered with a fine glabrate indumentum.

##### Type.

COLOMBIA. Antioquia: Municipio Urrao. Corregimiento La Encarnación, vereda Calles, Parque Nacional Natural Las Orquídeas, finca de Alfonso Pino, en la divisoria de aguas entre las quebradas La Virola y El Bosque, al noroccidente de la cabaña de Calles, 6°31'35"N; 76°15'50"W, 1450–1470 m, 27 Jan 2011 (fl, fr), *Paola Pedraza-Peñalosa et al. 2016* (holotype: COL!; isotypes, CAS!, CUVC, E!, HUA, MO!, NY! [NY02058404], PSO).

##### Description.

Epiphytic *shrub*, lianoid; stems brown-grey, terete, glabrous, bark smooth; twigs terete, smooth, glabrous. Axillary buds compressed; prophylls 2, valvate, lanceolate, inconspicuous, 2.5–4.5 mm long, margin eciliate, apex acuminate, abaxially puberulous, the hairs inconspicuous, ca. <0.2 mm long, eglandular and unicellular (all indumentum composed of this type of hairs except when indicated). *Leaves* alternate but apparently pseudoverticillate, originating in clusters of (2–)5–8 leaves separated by leafless sections 11.5–17.5 cm long; petiole terete, pulvinate, 3–6 mm long, glabrate; lamina coriaceous, elliptic, sometimes oblanceolate, (14–)18.7–28 × (3–)5–8 cm, base subcordate, margin entire and eciliate, apex acuminate, adaxially glabrous, abaxially glabrate (but appearing glabrous), the hairs inconspicuous, (< 0.2 mm long), caducous but with persistent bases that are red and swollen, apparently multicellular and glandular; laminar glands inconspicuous, basal, rounded; venation plinerved (acrodromous), suprabasal, with 3–4 visible orders (consistently well-marked up to 3^th^ order) in dry specimens, prominent lateral veins 2(–3) per side, subopposite or alternate, concentrated in the basal third, ascending, brochidodromous, midvein and secondaries adaxially impressed and abaxially raised. *Inflorescence* a 4–6-flowered raceme, more than one raceme arising from an axillary pad (pincushion like), often cauliflorous; inflorescence and floral bracts alike, persistent, chartaceous, ovate, 0.5–2.6 × 0.8–1.2 mm, margin entire and ciliolate, the hairs inconspicuous and eglandular, apex acute to acuminate, adaxially glabrous, abaxially glabrate, venation obscure; rachis orange, 4–8.5 mm long, glabrate, warts abundant and minute; pedicel dark or lightly orange, articulate with calyx, 1.9–2.3 cm long (3 cm when fruiting), basally (1–1.2 mm diam.) less than half the diameter of apex (3–4.5 mm) (*in vivo* 2 mm basally, 5–7 mm apically), with the apex becoming discoid and almost as wide as the calyx tube, glabrate, minute warts present at very base both *in vivo* and when dry; bracteoles 2, basal, supopposite to opposite, chartaceous, ovate, 2–2.4 × 0.6–0.8 mm, margin entire and ciliolate, the hairs inconspicuous, caducous and eglandular, apex acuminate, glabrous on both sides, venation obscure. *Flowers* 5-merous (some calyces 6-merous), actinomorphic, diplostemonous. Calyx dark or light orange, campanulate, with a marked transversal constriction between limb and tube, inconspicuously 5-angled, the angles alternating with lobes, conspicuously ribbed in the tube and with softer ribs in the limb, 4.1–5.8 mm long (6–7.2 mm *in vivo*), glabrate, the hairs inconspicuous, <0.1 mm long; tube oblate, 2–2.2 mm long (2.5–3 mm *in vivo*); limb slightly flaring, 2.1–3.6 mm long (3.5–4.7 mm *in vivo*); lobes deltate, 1–2 × 3.5–4.5 mm long (1.6–2 × 5 mm *in vivo*), margin entire, eglandular, and eciliate, apex acute; sinuses practically flat (broadly U-shaped *in vivo*); aestivation valvate. Corolla basal two thirds orange, apical third green, fleshy, not bistratose, obconic, 3.2–4 cm long (3.4–4.4 cm *in vivo*), 1–1.2 cm diam. (1–1.1 cm *in vivo*), 3 mm wide at throat (4.7–5.5 mm *in vivo*), inconspicuously 5-angled, glabrate without, the hairs inconspicuous, <0.1 mm long, glabrous within; lobes ovate, 1.5–2 × 1–2 mm, but sinuses often further tearing toward end of anthesis and the lobes then oblong and 4.8–7 × 1–2 mm, apex acute, not strongly reflexed at maturity; aestivation valvate. *Stamens* 10, dimorphic, staminal cycles with different anther lengths and dehiscence orientation, included, not adherent to corolla. Long stamens 10–11.2 mm long; filaments connate into a tube, straight, 3.5–4 mm long, glabrous or distally glabrate on the abaxial side, the hairs a handful, inconspicuous, multicellular and apparently eglandular; anthers 9–9.5 mm long, slightly prognathous, narrowing at base and widening at apex, without a clear distinction between tubules and thecae; thecae 6–6.5 mm long, without basal appendage, minutely papillate at least basally; tubules 2, free, turned inwards like bull’s horns, 3 mm long, smooth, dehiscing by latrorse elliptical slits 3 mm long, distal margin ornamented with small and irregular lobes. Short stamens 9–10 mm long, same shape, indumentum and features as long stamens except when indicated; filaments 3.5–4 mm long; anthers 8–8.5 mm long; thecae 5.5 mm long; tubules pointing upwards and without space between them, 2.5–3 mm long, dehiscing by introrse elliptical slits 2.5–3 mm long. Nectary not pulvinate, slightly concave to flat, glabrous; style white, 2.6–3.7 cm long, included; stigma punctiform. *Berry* cream-colored and ribbed when immature, turning purple at maturity.

**Figure 9.**
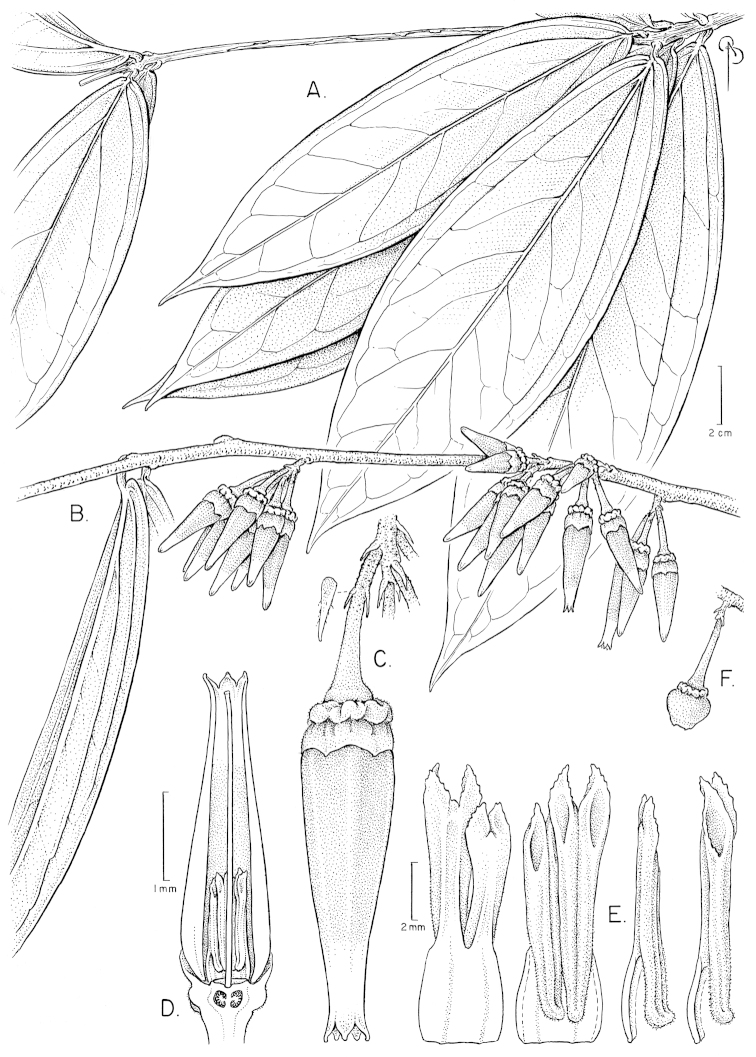
Illustration of *Satyria orquidiensis*. **A** Branch with clustered leaves **B** Branch with inflorescences **C** Flower attached to the rachis with detail of a bracteole **D** Longitudinal section of a flower **E** Stamens in abaxial, adaxial, and lateral views, respectively **F** Fruit. [Drawn from the type, *P. Pedraza-Peñalosa et al. 2436* and *2447*.]

**Figure 10.**
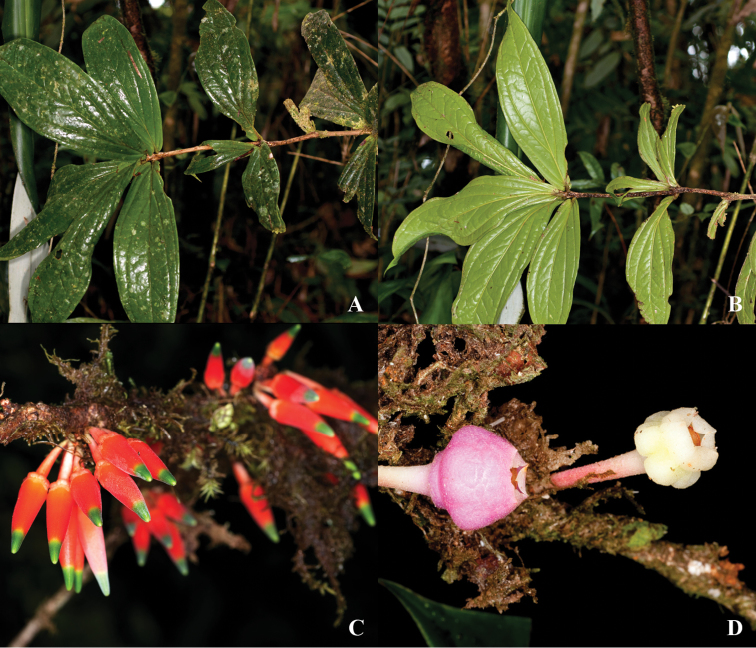
*Satyria orquidiensis*. **A** Branch with clustered leaves, adaxial side **B** Branch with clustered leaves, abaxial side **C** Cauliflorous inflorescences **D** Nearly mature (right) and immature fruits (left). [Photos by P. Pedraza-Peñalosa.]

**Figure 11.**
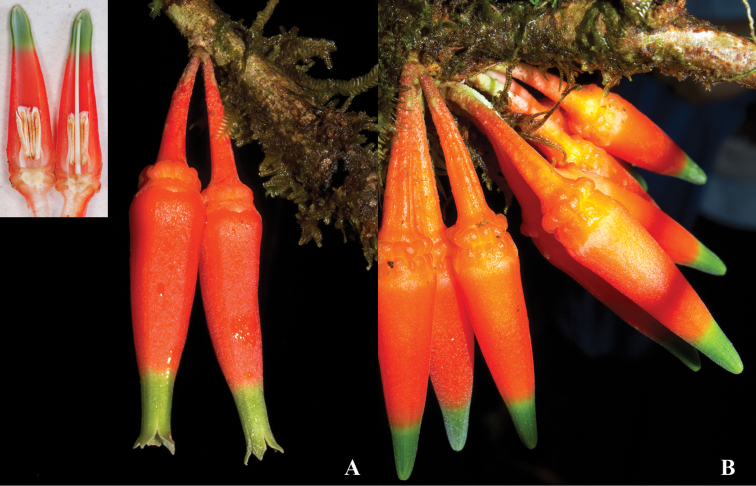
*Satyria orquidiensis*. **A** Flowers at anthesis with detail of a longitudinal section **B** Close-up of flowers showing the ornamented calyces. [Photos by P. Pedraza-Peñalosa.]

##### Distribution and ecology.

*Satyria orquidiensis* is endemic to Antioquia (Colombia) and it only known from collections from Las Orquídeas National Park. It is known to flower in January and fruit in January and July. This species grows in the canopy of humid premontane and montane forests at 880-1470 m, but it is possible that it could be found at lower altitudes as well.

##### Etymology.

Named after Las Orquídeas National Park (Colombia), where the species is endemic to.

##### Preliminary conservation status.

*Satyria orquidiensis* it is only known by specimens collected in Las Orquídeas National Park. It is commonly observed in conserved premontane and montane forests, but because it is a liana normally found high in the canopy, only a few collections exits. Because the observed abundance within the protected area I consider this species of least concern.

##### Discussion.

Morphologically, *Satyria orquidiensis* and *Satyria pterocalyx* stand out within the genus and have more similarities among themselves than with other species; these are the only species in the genus with conspicuous wings and/or ribs on their calyces. Although their large corollas share similar colors and obconic shape, they can be easily differentiated because *Satyria orquidiensis* has corollas inconspicuously 5-angled (vs. terete in *Satyria pterocalyx*), orange with green lobes (vs. red-orange with the tube and lobes green-whitish) that gradually decrease in diameter toward the lobes (vs. dramatically constricted at the throat which is elongated into a tube ca. 8 mm long). Also, the dark or light orange (vs. light green) calyces of *Satyria orquidiensis* are conspicuously ribbed on the tube and sharply constricted between the limb and tube (vs. calyces 5-winged, tube with two basal lobes in each of the facets demarked by the wings, not transversally constricted). Vegetetatively, these two new species are even more distinct as *Satyria orquidiensis* has leaves that are clustered and seemingly verticillate (vs. not clustered in *Satyria pterocalyx*), larger [(14–)18.7–28 cm long vs. 14–18 cm], basally subcordate (vs. obtuse or cuneate), apically acute to acuminate (long acuminate, acumen 1.8–2.8 cm long), and with inconspicuous basal laminar glands (vs. absent).

##### Specimens examined.

**COLOMBIA. Antioquia:** Municipio Frontino, Vereda Venados Abajo, Parque Nacional Natural Las Orquídeas, sector Venados, sitio La Miquera, 6°32'28.1"N; 76°18'05.3"W, 1000–1030 m, 27 Jul 2011 (fr), *P. Pedraza-Peñalosa et al. 2436* [COL!, NY! (2 sheets)]; Municipio Frontino, Vereda Venados Abajo, Parque Nacional Natural Las Orquídeas, sector Venados, sitio La Esperanza, cuenca de la quebrada Arenales, 6°42'06.8"N; 76°18'46.03"W, 880-920 m, 29 Jul 2011 (fl), *P. Pedraza-Peñalosa et al. 2447* (COL!, MO!, NY!).

#### Satyria pterocalyx

Taxon classificationPlantaeEricalesEricaceae

Pedraza sp. nov.

urn:lsid:ipni.org:names:77146699-1

Figures 12 , 13

##### Diagnosis.

*Satyria pterocalyx* can be easily differentiated among all other species in the genus by the following combination of characters. Its leaves are elliptic, often slightly asymmetric with the apex slanted to one side, large (14–18 cm long) and apically long acuminate (acumen 1.8–2.8 cm long). Its calyces are light green, 5-winged, with each of the facets of the calyx demarked by the wings containing two basal lobes that together look like an inverted m. In dry specimens, the pedicels have inconspicuous warts. Its corollas are very characteristic, terete, obconic and noticeably constricted at the throat, which is then elongated into a tube ca. 8 mm long; the corolla is red-orange with the tube and lobes green-whitish.

##### Type.

COLOMBIA. Antioquia: Municipio Urrao, Vereda La Magdalena, camino de herradura desde La Magdalena al Río Ocaidó, pasando por el Alto del Caballo, cuencas ríos Orougo, Orougito y Ocaidó, 6°14'05"–6°16'55"N; 76°13'24"–76°15'14"W, 1730–2150 m, 13 Dec 2007 (fl), *P. Pedraza-Peñalosa, J. Betancur, F. Gómez & O. Laverde 1755* (holotype: COL!; isotypes: HUA!, MO!, NY!).

##### Description.

Epiphytic *shrub*, lianoid; stems brown-grey, terete, glabrous, bark smooth; twigs subterete, smooth, glabrate, the hairs inconspicuous (< 0.1 mm long), unicellular and eglandular (all indumentum composed of this type of hairs except when indicated). Axillary buds compressed; prophylls 2, valvate, lanceolate, conspicuous, 3.6–4.1 mm long, margin eciliate, apex acuminate, glabrous. *Leaves* alternate; petiole terete, not pulvinate, 6–8 mm long, glabrescent; lamina coriaceous, elliptic, often slightly asymmetric with the apex slanted to one side, 14–18 × 5.8–7.3 cm, base obtuse or cuneate, margin entire and eciliate, apex long acuminate (acumen 1.8–2.8 cm long), adaxially glabrous, abaxially glabrate, the hairs inconspicuous, (< 0.1 mm long), multicellular and eglandular; laminar glands absent; venation plinerved (acrodromous), suprabasal, with 3–4 visible orders in dry specimens, prominent lateral veins 2(–3) per side, subopposite, concentrated in the basal third, ascending, brochidodromous, midvein and secondaries adaxially impressed and abaxially raised. *Inflorescence* a axillary, solitary, raceme with at least 2 flowers, cauliflorus; inflorescence bracts, floral bract, and bracteoles alike, persistent, chartaceous, ovate, 1–1.6 × 0.5–1 mm, margin entire and ciliolate, the hairs inconspicuous, caducous and eglandular, apex acute to acuminate, glabrous on both sides, venation obscure; rachis green, 5–12 mm long, glabrous; pedicel orange, articulate with calyx, 2.6–3 cm long, basally less than half the diameter of apex (*in vivo* 1.5 mm vs. 4.5 mm, respectively), glabrescent, with inconspicuous warts basally (not evident *in vivo*); bracteoles 2, basal, supopposite to alternate. *Flowers* 5-merous, actinomorphic, diplostemonous. Calyx light green, oblate (more or less campanulate when dry), 3.8–5.6(–7.7) mm long (6.2–6.5 mm *in vivo*), 5-winged, the wings alternating with lobes, minutely puberulous; tube oblate, 3–3.2(–5.2) mm long (3.5–4.1 mm *in vivo*), the base conspicuously lobed, each facet of the calyx demarked by the wings contains two basal lobes that together look like an inverted m; limb more or less erect, 1.6–2(–2.5) mm long (2.4–3.3 mm *in vivo*); lobes deltate, 0.8–1 × 3–3.5 mm long (0.5–1.2 × 4–6 mm *in vivo*), margin entire, eglandular, and eciliate, apex obtuse; sinuses obtuse (U-shaped) to almost flat; aestivation unknown. Corolla red-orange with the tube and lobes green-whitish, fleshy, bistratose, obconic and noticeably constricted at the throat which is elongated into a tube (ca. 8 mm long), terete, 2.8–3(–4) cm long, 1.2–1.3 cm diam., 2.7–3 mm wide at throat (4 mm *in vivo*), inconspicuously puberulous without with a combination of hairs minute (< 0.5 mm long), eglandular and unicellular, along with a few hairs eglandular and multicellular, glabrous within; lobes deltate, 1.1 × 1.2–1.5 mm (lanceolate, 4.3 × 2 mm *in vivo*), apex acute, not strongly reflexed at maturity; aestivation unknown. *Stamens* 10 (all measurements *in vivo*), dimorphic, staminal cycles with different anther lengths and dehiscence orientation, included, not adherent to corolla. Long stamens 9.8–10.8 mm long; filaments connate at base, straight, 3–4 mm long, glabrate, the hairs inconspicuous and eglandular, the marginal ones unicellular, the abaxial ones multicellular, very scarce and distally concentrated, adaxial side glabrous; anthers 8.6–10.1 mm long, narrowing at base and widening at apex, without a clear distinction between tubules and thecae; thecae 5.9–7.1 mm long, without basal appendage, papillate at base, smooth at apex; tubules 2, free, pointing upwards, 2.5–3 mm long, smooth, dehiscing by latrorse elliptical slits 2.2–2.5 mm long, abaxial side and margin ornamented with irregular epidermal projections. Short stamens 8.3–9.5 mm long, same shapes, indumentum and features as long stamens except when indicated; filaments 3–3.5 mm long; anthers 7.5–9.4 mm long; thecae 5.2–6.4 mm long; tubules 2.3–3 mm long, dehiscing by introrse elliptical slits 2.2–2.5 mm long. Nectary pulvinate, not too prominent, glabrous; style 2.8–3.2 cm long, included; stigma discoid. *Berry* unknown.

**Figure 12.**
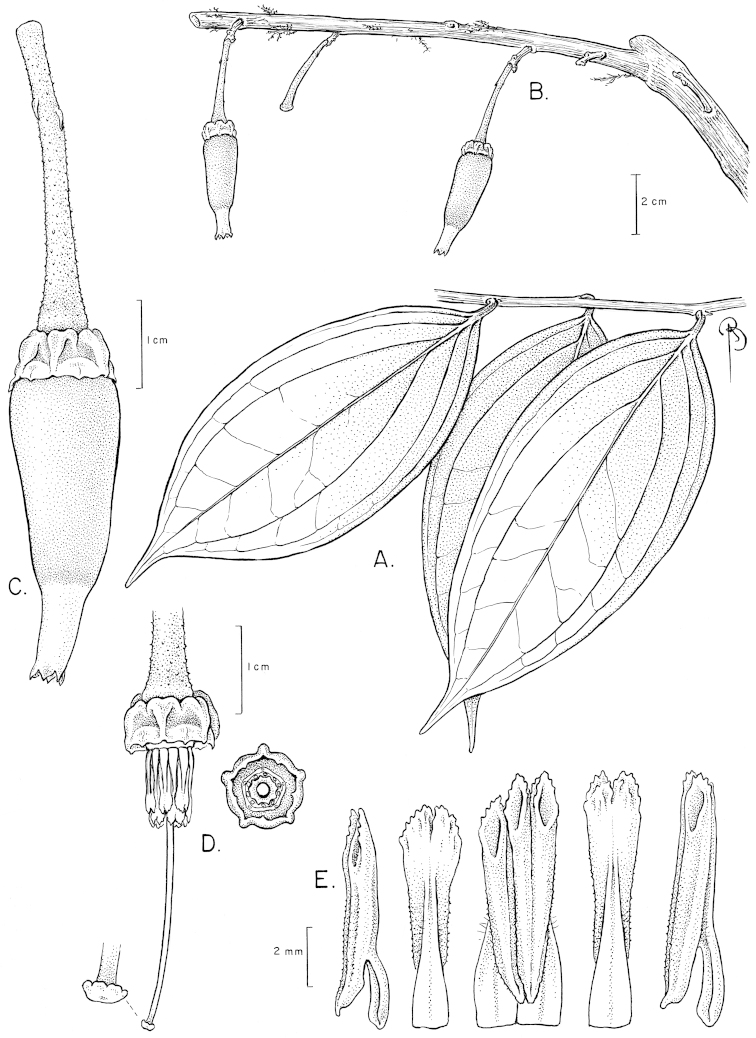
Illustration of *Satyria pterocalyx*. **A** Branch with leaves **B** Branch with flowers **C** Flower with pedicel **D** Flower with the corolla removed to show the stamens arranged around the style; detail of the nectary from above and of the stigma **E** Stamens in abaxial, adaxial, and lateral views. [Drawn from the type.]

**Figure 13.**
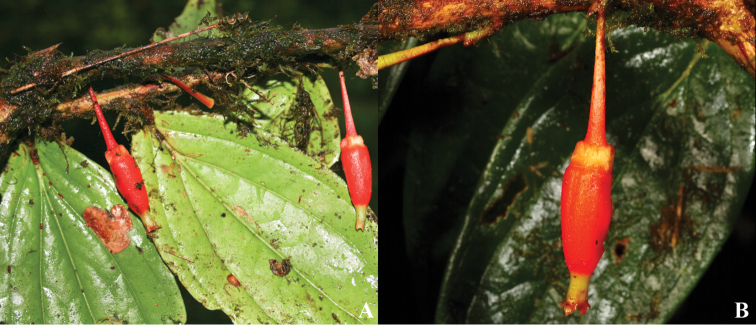
*Satyria pterocalyx*. **A** Branch with leaves and flowers **B** Flowers at anthesis. [Photos by P. Pedraza-Peñalosa.]

##### Distribution and ecology.

*Satyria pterocalyx* is restricted to the biologically rich montane forests of Western Colombia (Antioquia, Choco). It is known to flower in December and January.

##### Etymology.

Species named after the rare winged calyces.

##### Preliminary conservation status.

*Satyria pterocalyx* is known from two localities far apart (from adjacent departamentos) that confer it a not so small geographic range. However, after botanizing for several years in Antioquia, this species remains only known by two individuals. Although collected a few miles from Las Orquídeas National Park, *Satyria pterocalyx* has not been found within the protected area. Currently, Colombian montane forest suffer from degradation product of human activities (agriculture, selective logging, livestock, mining, etc.), therefore I consider this species vulnerable due to its perceived scarcity and current habitat threats.

##### Discussion.

The morphological differences and similarities between *Satyria pterocalyx* and *Satyria orquidiensis* are discussed under the latter.

##### Specimens examined.

**COLOMBIA. Choco:** Alto del Buey, 1200–1800 m, 8 Jan 1973 (fl), *A. Gentry & E. Forero 7311* (NY!).

## Supplementary Material

XML Treatment for Psammisia pinnata

XML Treatment for Psammisia pseudoverticillata

XML Treatment for Psammisia sophiae

XML Treatment for Satyria orquidiensis

XML Treatment for Satyria pterocalyx
